# Assembling the Current Pieces: The Puzzle of RNA-Mediated Regulation in *Staphylococcus aureus*

**DOI:** 10.3389/fmicb.2021.706690

**Published:** 2021-07-21

**Authors:** Laura Barrientos, Noémie Mercier, David Lalaouna, Isabelle Caldelari

**Affiliations:** Université de Strasbourg, CNRS, Architecture et Réactivité de l’ARN, UPR 9002, Strasbourg, France

**Keywords:** regulatory RNA, interconnected network, *Staphylococcus aureus*, virulence, metabolism

## Abstract

The success of the major opportunistic human *Staphylococcus aureus* relies on the production of numerous virulence factors, which allow rapid colonization and dissemination in any tissues. Indeed, regulation of its virulence is multifactorial, and based on the production of transcriptional factors, two-component systems (TCS) and small regulatory RNAs (sRNAs). Advances in high-throughput sequencing technologies have unveiled the existence of hundreds of potential RNAs with regulatory functions, but only a fraction of which have been validated *in vivo*. These discoveries have modified our thinking and understanding of bacterial physiology and virulence fitness by placing sRNAs, alongside transcriptional regulators, at the center of complex and intertwined regulatory networks that allow *S. aureus* to rapidly adapt to the environmental cues present at infection sites. In this review, we describe the recently acquired knowledge of characterized regulatory RNAs in *S. aureus* that are associated with metal starvation, nutrient availability, stress responses and virulence. These findings highlight the importance of sRNAs for the comprehension of *S. aureus* infection processes while raising questions about the interplay between these key regulators and the pathways they control.

## Introduction

*Staphylococcus aureus* is a major opportunistic human pathogen capable of causing an extensive array of human infections, ranging from easy-treatable sinusitis to life-threatening endocarditis or septicemia. Its versatility in colonizing diverse human organs relies on the temporally coordinated expression of numerous virulence factors allowing the bacterium to adhere, invade and disseminate into host tissues. Regulation of virulence factors expression is conducted by two-component systems (TCS), transcriptional regulators and in particular small regulatory RNAs (sRNAs). These include *cis*-acting RNAs such as antisense RNAs or riboswitches, and *trans*-acting RNAs (Carrier et al., 2018; Jørgensen et al., 2020).

The latter generally control multiple messenger RNAs, especially by targeting their Shine–Dalgarno (SD) sequence, which results in translational repression and/or stability modulation. Indeed, many staphylococcal sRNAs contain a characteristic C-rich sequence complementary to the SD sequence of targeted mRNAs (Geissmann et al., 2009). In many bacteria, sRNA:mRNA interactions are mediated by the chaperones Hfq or ProQ. However, the role of Hfq in *S. aureus* is still controversial and ProQ is not present (Christopoulou and Granneman, 2021). Even though staphylococcal Hfq is able to bind some sRNAs *in vivo* and *in vitro*, it does not facilitate sRNA-mRNA interactions (Bohn et al., 2007). In addition, its deletion has no effect on sRNA-mediated regulation and did not present any specific phenotype. The dispensability of Hfq may result from longer and, consequently more stable, sRNA-mRNA duplexes than the ones requiring Hfq in *Escherichia coli* (Jousselin et al., 2009).

The functions of sRNAs in gene regulation and physiological responses in bacteria are now well established. Their ability to regulate specific metabolic pathways and stress responses makes them ideal candidates to regulate virulence in pathogenic bacteria. Indeed, in *S. aureus* the bi-functional sRNA RNAIII is the main intracellular effector of the quorum sensing system and controls temporal expression of virulence genes, in addition to containing the open reading frame (ORF) for the phenol soluble modulin (PSM) hemolysin delta (Bronesky et al., 2016). Besides, RNAIII, RsaA, SprC, SprD; Teg49 and SSR42 contribute to different facets of virulence regulation in animal models of infection (Desgranges et al., 2019).

## Discovery of sRNA in *Staphylococcus aureus*

The use of predictive bioinformatic searches, microarrays and expression studies led to the discovery of the first sRNAs in *S. aureus* (Pichon and Felden, 2005; Geissmann et al., 2009; Nielsen et al., 2011). Then, the advances in high-throughput sequencing technologies opened the door to a whole new era in the small RNA field (Desgranges et al., 2020). It not only helped and accelerated the discovery of further RNAs with regulatory functions in *S. aureus* (Abu-Qatouseh et al., 2010; Beaume et al., 2010; Bohn et al., 2010; Howden et al., 2013; Carroll et al., 2016; Mäder et al., 2016), but also facilitated their characterization by promoting global analyses of transcriptional changes they induce. sRNAs are commonly encoded in intergenic regions or are originated from 3′ or 5′-UTR of mRNAs and are associated to the regulation of numerous metabolic pathways and virulence. Accessibility of these sequencing techniques accumulated huge transcriptomic data. However, the lack of a consensual and fully annotated *S. aureus* genome added to a missing unified sRNA nomenclature led to numerous redundancies and misannotated sRNAs. To overcome this matter, Sassi et al. (2015) designed the *Staphylococcus* Regulatory RNA Database (SRD) which provides a simple and non-redundant list of sRNAs identified in *S. aureus*. Sequences of transcribed sRNAs were compiled from various RNAseq analyses to yield a non-redundant catalog of ca. 500 sRNAs assigned with a single identifier. This list is drastically reduced to 50 when only *trans-*acting sRNAs are considered (Liu et al., 2018). Unfortunately, most putative 5′/3′-UTR-derived sRNAs are discarded here. Very recently, Carroll’s team re-analyzed published RNAseq and ribosome profiling data scrutinizing the expression and stability or capacities to encode peptides of 303 sRNAs in different conditions, showing their diversity in behavior and functions (Sorensen et al., 2020). Altogether, these studies raise issues about the poor annotation of staphylococcal genome concerning sRNAs. Furthermore, the effort of the scientific community in sequencing genomes of many staphylococcal isolates will considerably improve it.

## Diving Into sRNA Networks

**FIGURE 1:**
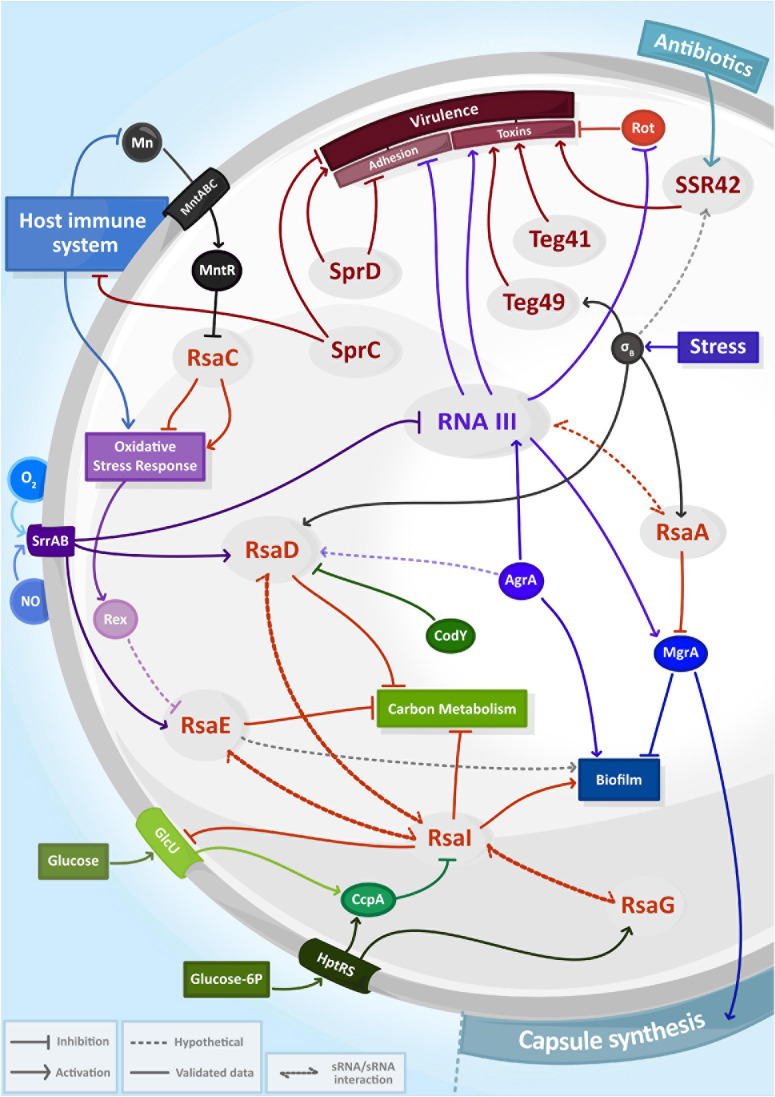
The complexity and entanglement of regulatory RNA (sRNA) networks in *Staphylococcus aureus*. Expression of sRNAs (in gray circles) are induced by environmental signals including antibiotics, host immune system responses, exposure to reactive species (NO, O_2_^–^) and nutrient availability. Together with transcriptional factors (solid circle) or two-component systems, sRNAs control capsule synthesis, biofilm production, carbon metabolism, oxidative stress response or virulence and then form intricate regulatory networks.

To unravel the functions of a newly identified sRNA, it is necessary to define its partners. The identification of RNA candidates as direct targets of sRNAs would provide hints of their roles and pathways, in which a specific sRNA might be involved. Several experimental techniques have been recently developed to characterize sRNA targetomes in bacteria, mostly based on the pull-down of chaperone proteins such as Hfq followed by sequencing of associated RNAs (Desgranges et al., 2020). In *S. aureus*, preference was given to a distinct approach, which relies on the co-purification of binding partners with a biotinylated/tagged sRNA. These methods called MAPS (Lalaouna et al., 2015; Mercier et al., 2021) and Hybrid-trap-seq (Rochat et al., 2018) have been used to determine the interactome of various sRNAs in *S. aureus*, generating ever more complex regulatory networks picturing many events: one sRNA involved in different pathways, several sRNA involved in the same pathway or sRNAs associated with one another (Figure 1). This has highlighted the complexity and intertwined nature of sRNA networks in *S. aureus*, which most certainly accounts for the versatility of this pathogen.

Overall, of the numerous sequences for potential sRNAs, only a small fraction has been experimentally confirmed and many more remain to be characterized. In this work we will review the current state of the art of the sRNA world from *S. aureus*, featuring those involved in virulence, nutrient availability, metal starvation and stress responses. We will focus predominantly on recent results deciphering functions of staphylococcal sRNAs, while others were extensively reviewed in Guillet et al. (2013), Tomasini et al. (2013), and Desgranges et al. (2019).

## The Quest of Power: A Larger Arsenal of sRNAs Regulating Virulence: Teg41 and SSR42

Besides the well described RNAIII, RsaA, Teg49 and SprC/D (Desgranges et al., 2019), two sRNAs, Teg41 and SSR42, appeared recently to regulate virulence in *S. aureus*. Teg41 is a 205 nt-long sRNA that is divergently transcribed from the locus encoding alpha phenol soluble modulins (αPSM), highly potent pore-forming toxins exhibiting cytolytic activity (Zapf et al., 2019). The deletion of 24 nts in its 3′ end is sufficient to lower αPSM production at the protein level, reduce hemolytic activity and attenuate virulence in a murine abscess model. Conversely, Teg41 overexpression enhances hemolytic activity by increasing αPSM protein levels. *In silico* predictions suggest the binding of Teg41 after the start codon of αPSM4, the 4th gene of the operon and most abundant αPSM. However, this interaction remains to be confirmed as well as the activation mechanism of *psmα4*. Several hypotheses are raised, such as a positive regulation where Teg41 would stabilize the PSM transcript to facilitate its translation or induce conformational changes to free the ribosome binding site (RBS) together with an unknown partner. This would be the first time that an sRNA has been directly linked to the regulation of αPSM, since only protein regulators such as MgrA or AgrA are known to regulate the transcription of mRNA encoding these toxins (Jiang et al., 2018). Interestingly, the *teg41* gene is restricted to *S. aureus* and the very closely related *Staphylococcus argenteus*, and this conservation seems to correlate with the presence of the αPSM locus. This suggests that both might be genetically linked (Zapf et al., 2019).

The 1,232 nt-long SSR42 belongs to the family of small stable RNAs (SSR), a group of regulatory RNAs induced and/or stabilized during stress-related conditions such as log-phase growth, heat/cold shock or stringent response (Anderson et al., 2006; Morrison et al., 2012). SSR42 is stabilized during stationary phase where it mostly represses expression of several virulence genes through an indirect yet undescribed mechanism, probably by regulating transcriptional regulators of these virulence factors (Morrison et al., 2012). This sRNA is also required for hemolysis and for virulence in a murine model of skin infection. SSR42 is located directly upstream and in an antiparallel orientation from the gene encoding Rsp, the repressor of surface proteins, a regulator of hemolysis that positively controls the production of *hla via* the *agr* system (Li et al., 2015). Rsp activates the expression of SSR42 in presence of antibiotics as oxacillin. Consecutively, SSR42 enhances hemolysis by acting indirectly on the *hla* promoter during stationary phase (Horn et al., 2018). SSR42 therefore places itself in line with regulators such as SaeR and SarA for transcriptional activation of *hla* and RNAIII for its translation. Besides, SSR42 stabilizes the *sae* transcript encoding the major transcriptional regulator of *hla* through a yet unknown mechanism (Horn et al., 2018). This suggests that modulation of *sae* transcript impacts Hla production. SSR42 therefore participates in the complex regulation of *hla* transcription in response to antibiotics even though the molecular mechanisms remain elusive.

## RsaC, the Missing Link Between Manganese Homeostasis and Oxidative Stress

RsaC length is highly variable across *S. aureus* isolates due to the presence of repeated sequences at its 5′ end and, consequently, ranges from 584 to 1,116 nts (Lalaouna et al., 2019). Remarkably, the characterization of RsaC provided the missing link between manganese homeostasis and oxidative stress response. The mutation of *mntABC*, coding for the major manganese ABC transporter, was previously reported as detrimental for Mn acquisition, but also for oxidative stress resistance (Handke et al., 2018). Lalaouna et al. (2019) demonstrated that RsaC derives from the 3′ untranslated region of *mntABC* after cleavage by the double-stranded ribonuclease RNase III. In manganese-limiting conditions, RsaC negatively regulates the non-essential Mn-containing superoxide dismutase A (SodA), which is involved in reactive oxygen species detoxification (O2^–^ to H_2_O_2_). Concurrently, RsaC favors the SodM-dependent oxidative stress response, an alternative SOD enzyme using either Fe or Mn as cofactor. Besides helping maintain the appropriate cellular Mn^2+^ concentration, it restores the ROS detoxification pathway and counteracts Mn sequestration by host immune cells.

Noteworthy, RsaC could also interconnect and balance various metallostasis systems (Fe and Zn) (Lalaouna et al., 2019), but is apparently not involved in the MntABC-mediated increased resistance to copper (Al-Tameemi et al., 2021).

## The Blurred Line Between Metabolism and Virulence: RsaD, RsaE, RsaI, and RsaG

RsaD, a 176 nt-long sRNA, is conserved in multiple staphylococcal species (Geissmann et al., 2009). It accumulates in the late exponential phase of growth and is highly expressed in strains with an active σB factor, which is responsible of the regulation of genes involved in stress response in *S. aureus*. Nonetheless, the exact mechanism of *rsaD* regulation by this factor remains uncertain. More recently, Augagneur et al. (2020) observed that expression of *rsaD* is repressed by CodY, a global regulator activated by branched amino-acids and GTP and regulating genes involved in primary metabolism and virulence (Brinsmade, 2017). The promoter region of *rsaD* contains a putative CodY binding motif, which was detected in at least 15 staphylococcal species, indicating that the regulation of *rsaD* by CodY is probably conserved (Augagneur et al., 2020). In addition, RsaD is activated during nitric oxide (NO) stress, sensed by the TCS SrrAB (Bronesky et al., 2019) and possibly by the quorum sensing system Agr (Marroquin et al., 2019). Thus, RsaD seems to assimilate multiple signals from the environment. To determine the physiological functions of RsaD, *in silico* analyses using RNA Predator, TargetRNA2 and IntaRNA identified *alsS*, which is positively regulated by CodY and whose product is involved in carbon metabolism, as a possible target. RsaD binds the RBS of *alsSD* mRNA through its C-rich region and inhibits its translation initiation, leading to a decrease in AlsS enzymatic activity (Augagneur et al., 2020). Thus, by repressing RsaD, CodY permits AlsS synthesis. When glucose is in excess, AlsSD (acetolactate synthase/decarboxylase) generates acetoin (a neutral-pH compound) from pyruvate and therefore protects bacteria from death due to acidification of the cytoplasm by increased acetate production. Then, in these conditions, RsaD must be repressed for survival. This work revealed the *trans-*acting regulatory activity of RsaD on at least one mRNA and highlights the balancing role of this sRNA in carbon overflow and its implications in cell survival (Augagneur et al., 2020). All these mechanisms by which RsaD might be regulated, integrate, and respond to different environmental cues remain to be unveiled. Its place in the complex regulatory RNA networks of *S. aureus* awaits to be established.

RsaE is a highly conserved sRNA among the Firmicute phylum. This striking conservation emphasizes the crucial role of RsaE in metabolism adaptation. First discovered in *S. aureus*, this 93 nt long sRNA is composed of two UCCCC motifs critical for its interaction with the RBS of its mRNA targets (Geissmann et al., 2009; Rochat et al., 2018). Its expression depends on the activation of the TCS SrrAB that responds to low oxygen concentration and NO exposure (Kinkel et al., 2013). A similar activation pattern is described in *B. subtilis* with its homolog RoxS (Durand et al., 2015). In addition, RoxS is repressed by the NAD + /NADH sensor Rex whose binding site is conserved, which suggests that Rex could fulfill a similar role in *S. aureus*.

RsaE is involved in the regulation of central metabolic pathways, in particularly by negatively regulating numerous enzymes of the TCA cycle and folate metabolism (Geissmann et al., 2009; Bohn et al., 2010; Rochat et al., 2018). Among its targets, RsaE inhibits the translation of *rocF* mRNA, which encodes an arginase responsible of converting arginine into ornithine (Rochat et al., 2018). Furthermore, the absence of RsaE stimulates growth rate in a medium containing exclusively 18 amino acids (all except glutamine and asparagine) as sole carbon sources, positioning RsaE as a major repressor of amino-acid catabolism.

Surprisingly, RsaE is processed in *S. epidermidis* and *B. subtilis* but apparently not in *S. aureus* (Rochat et al., 2018). In *S. epidermidis*, the processed form of RsaE (RsaEp) expands its targetome with the transcripts of the main biofilm repressor IcaR or of the succinyl-CoA synthetase SucCD, an enzyme involved in TCA cycle (Schoenfelder et al., 2019). Interestingly, both mRNAs only interact with RsaEp. In *B. subtilis*, RNase Y is responsible of RoxS cleavage, however, in a *S. aureus* RNase Y mutant strain, the levels of RsaE or its targets are not impacted (Marincola et al., 2012). Still, a processed RsaE could act on yet unknown mRNAs. Altogether, RsaE interferes with the TCA cycle by directly inhibiting related enzymes and by limiting the production of amino-acid alternative substrates. It has been suggested in *S. aureus* and in *B. subtilis* that RsaE balances NAD + /NADH ratio when environmental stimuli (such as O_2_ concentration or NO exposure) trigger a metabolism slowdown (Durand et al., 2015).

Additionally, RsaE interacts with another sRNA named RsaI involved in sugar metabolism control (Rochat et al., 2018; Bronesky et al., 2019), that could potentially connect the regulation network of both sRNAs. It cannot be excluded that RsaE or RsaI could behave as an sRNA sponge of one another, promoting the decay or sequestration of the other partner. RsaI is a 144 nt long sRNA conserved among the *Staphylococcacea* family. The expression of RsaI is repressed by the catabolite control protein A (CcpA) in presence of glucose (Bronesky et al., 2019). When glucose has been metabolized, RsaI inhibits the translation of the main glucose uptake protein GlcU and activates enzymes acting in glucose fermentation. On the other hand, RsaI represses FN3K expression, a protein protecting the bacterium from the damages caused by high glucose concentration, positioning RsaI at the core of regulatory pathways of sugar metabolism. Interestingly, RsaI binds the 3′UTR of *icaR* mRNA and thus promotes biofilm formation by a mechanism which is still unsolved (Bronesky et al., 2019). To note, the *icaR* messenger was pulled out with RsaE *in vitro*, and sequencing suggested that it interacts with the 5′UTR of *icaR* such as in *S. epidermidis* (Rochat et al., 2018). Knowing that RsaE and RsaI form a duplex, further experiments would be necessary to decipher the intricacy of regulatory lines among all these RNAs.

In addition to RsaE, RsaD (see above) and the glucose-6-phosphate induced sRNA RsaG were enriched with RsaI in MAPS, but the relevant significance of these interactions has not been explained yet (Bronesky et al., 2019). Interestingly, it has been suggested that RsaI promotes the expression of NO detoxification or anaerobic metabolism enzymes as an indirect effect of its interaction with RsaE, RsaD and RsaG. Nevertheless, shared signals and targets between these sRNAs imply tight connections and that all these regulatory networks would rationally impact each other at different levels, connecting sugar metabolism and stress responses.

## Conclusion

In the recent years, several tools were developed to decipher the functions of staphylococcal sRNAs. They revealed that sRNAs sense and reply to different environmental stimuli and that they mostly control mRNA translation to remodel metabolomic pathways to adapt and survive in harsh environments conditions.

The more the identified sRNAs are studied, the clearer it becomes that there is no isolated node in regulatory network or pathway, but a myriad of interconnections that we are only at the beginning to acknowledge. Exciting discoveries await for us in the years to come, as all these interrelationships will be straightened out and a clearer map of sRNA interactions will be drawn.

In the meantime, many questions about the sRNA world in *S. aureus* remain to be addressed. The significance of RNA-binding proteins in all these networks is still very uncertain, besides the established role of RNase III in sRNA maturation and target degradation. However, there could be holes in the puzzle that might be filled in by some of these proteins, which may help explaining unsolved sRNA-dependent mechanisms of action.

The study of the complex regulatory networks of *S. aureus*, in which sRNAs are at the center, is undoubtedly essential for understanding its virulence and adaptation mechanisms and will ultimately guide us in the design of treatments to fight this pathogen.

## Author Contributions

LB, NM, DL, and IC contributed to the manuscript writing. All authors contributed to the article and approved the submitted version.

## Conflict of Interest

The authors declare that the research was conducted in the absence of any commercial or financial relationships that could be construed as a potential conflict of interest.
